# Current Undergraduate Dental Implantology Teaching in UK

**DOI:** 10.3390/dj10070127

**Published:** 2022-07-05

**Authors:** Arminder Hare, Samantha Bird, Simon Wright, Cemal Ucer, Rabia Sannam Khan

**Affiliations:** 1 School of Health and Society, University of Salford, Salford M5 4WT, UK; ash456m@aol.com (A.H.); s.l.bird3@salford.ac.uk (S.B.); 2 ICE Postgraduate Dental Institute and Hospital, 24 Furness Quay, Salford M50 3XZ, UK; profwright@glencairndental.co.uk (S.W.); cemalucer@me.com (C.U.)

**Keywords:** dental implants, dental undergraduate, education, curriculum, barriers

## Abstract

Dental Implants are a popular treatment option for tooth replacement, with documented long-term success and survival rates of more than 95% over a period of 10 years. However, incorporating dental implantology into an undergraduate dental curriculum has issues associated. Therefore, the aim of this research was to examine and evaluate current undergraduate dental implantology education in the UK, investigate the amount of time allocated to this subject and analyse the barriers that are currently impeding the development of the programmes. An online questionnaire hosted by Online Surveys was designed, piloted, and sent to 16 dental schools providing undergraduate education in the UK. Ethical approval was gained from The University of Salford to conduct the study. Out of the 16 dental schools contacted, eight questionnaire responses were received, hence a response rate of 50% was achieved. The hours dedicated to the implant teaching programme varied from 3 h to 25 h, with a mean average of 11 h. It was identified from the results that no teaching of dental implantology was conducted in year 2; 12% of the schools responded that the subject was taught in year 1, 37% in year 3, 75% in year 4 and 50% in year 5. The methods used to deliver the programme were mainly lecture-based teaching, with only one dental school allowing students to place implants on patients. The main barriers to progression of the programme were financial (75%), followed by time limitations imposed by the curriculum (37%) and liability insurance (37%). **However, there appears to be a consensus that further training beyond bachelor’s degree level is required to teach implantology effectively.**

## 1. Introduction

The importance of implant dentistry in undergraduate education has become more widely recognised. It is necessary to provide new graduates with a specific degree of competency in this area, in order to prepare them for general practice [1]. Difficulty in incorporating additional content into an already overburdened curriculum is often used to justify the lack of emphasis on implant dentistry in undergraduate education [2,3]. Implant therapy may not be a priority for certain institutions and may be viewed as an advanced subject, only to be taught in postgraduate education [4]. Dental implants are widely regarded as an important treatment option for replacing missing teeth, as concluded in a meeting of the Association for Dental Education in Europe (ADEE) in 2008. This was the European consensus meeting and it was established that at the undergraduate level, training should incorporate diagnostic, surgical and therapeutic procedures in implant dentistry university education [5].

Blum (2008) conducted a study to examine the teaching of implant dentistry in the undergraduate curriculum of dental schools in the UK. The survey comprised of a questionnaire sent to all undergraduate dental schools in the UK [6]. A 100% response rate was achieved and the results showed all dental schools in the UK included dental implantology in their undergraduate curriculum. However, there were significant differences in the content and delivery at that time. Five years after the European consensus meeting in 2008, Koole and Bruyn studied the status of university teaching of implant dentistry and the effects of the guidelines set in 2008 for teaching and evaluation. The aim of the report was to define potential directions in the discipline’s education. Implant dentistry was becoming more integrated into undergraduate dental education. Moreover, it was determined that further changes were still required at all educational levels, in order to match the clinical training with the population’s growing care needs [7].

A more recent study by Chin, Lynch et al., (2018) assessed the status of implant teaching within undergraduate schools in the UK and Ireland. An online questionnaire investigating current and (potential) possible developments in dental implantology education was created. This was then distributed to heads of restorative dentistry departments in each of the 18 dental schools in the UK and Ireland that offer undergraduate dental programmes [8]. All of the schools that responded offered implant training to their students. The majority of schools provided students with direct clinical training in treatment preparation (*n* = 13), but not in implant restoration (*n* = 5) or implant placement (*n* = 1). Support and a lack of available time in the curriculum were the two main obstacles to introducing and expanding the dental undergraduate implant programme. The study concluded that, although the volume of implant teaching in the UK and Ireland had increased significantly, implant education in dental undergraduate schools needed to be further developed and improved, especially in terms of providing direct clinical experience [2]. This would ensure that newly trained dentists begin practice with a higher degree of experience in this area and are better prepared to practice independently [9]. The study did not investigate the years in which implantology was taught, or if there were training programmes to prepare staff to teach implantology to students.

The current study set out to assess the changes in curriculum of UK dental schools since 2008. Further to this, it was designed to assess if there had been more alignment in the curricula set by the universities [10]. In addition, the report investigates the factors which limit the progression of teaching implantology. The General Dental Council (GDC) recognises that the concept of replacing missing teeth with dental implants is an essential skill for a newly trained dentist. Students should be able to see implant procedures performed during their undergraduate programme, as well as observe dental implants being preserved in stable tissue (GDC 2002). Hence, implant dentistry must feature within the undergraduate dental curriculum.

The aim of this study was to examine and evaluate current undergraduate dental implantology education in the UK. We also investigated the time allocated to implant education and any barriers that were impeding the development of the programmes.

## 2. Materials and Methods

A cross-sectional study with an online questionnaire hosted by Online Surveys [6] was designed, piloted and sent to the 16 dental schools providing undergraduate dental education in the UK. The questionnaire was distributed with a cover letter in February 2021 to the programme administrators/coordinators and heads of undergraduate education or the responsible individual for coordination of teaching of dental implantology. The questionnaire contained both closed and open questions, to allow for comparisons between programmes and a more in depth understanding of the design, facilitators and barriers to dental implantology education at undergraduate level.

Face validity, readability, clarity and comprehensiveness of the questionnaire was assessed through pilot testing [2] and agreement was achieved by reviewers. Questions were designed in line with previous studies investigating implantology in dental education in the UK [11].

The questionnaire asked in which years implant dentistry is taught; how time is divided between theoretical, practical, and clinical practice; how the curriculum content related to implantology and what were perceived as the barriers and facilitators for the progression of implantology teaching.

Respondents were asked to select from options provided on year taught (between 1–5), teaching methods utilised, teaching aids, the format of practical teaching, procedures performed by undergraduates, topics covered, how implantology is assessed, training for staff, and barriers. Respondents were given options to add further or other answers to the closed questions. These responses were analysed with Microsoft Excel and GraphPad Prism to describe findings.

Open questions, analysed thematically, Refs. [12,13,14] elicited detailed responses on plans to change hours allocated to the teaching of implantology.

## 3. Results

Out of 16 dental schools in the UK, eight questionnaire responses were received, hence a response rate of 50% was achieved. 100% of the questions were answered. The results of the questionnaire were presented in the form of descriptive analysis. The open-end question responses from the questionnaire were presented as direct quotes. These were subjected to thematic analysis in an attempt to uncover patterns and themes, subthemes and codes for educational and clinical aspects.

**Figure 1:**
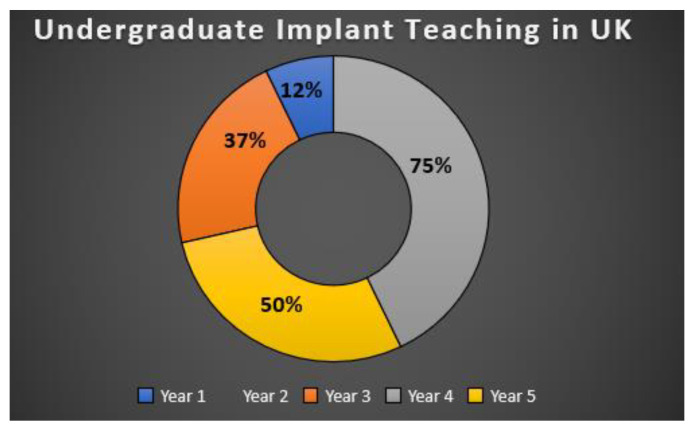
Pie chart shows the percentage of dental implant teaching in UK taught in 5 years of undergraduate dentistry.

The results showed that no teaching of dental implantology was conducted in year 2 of any dental school. 12% of the eight schools who responded taught it in year 1, 37% in year 3, 75% in year 4 and 50% had teaching in the curriculum in year 5 (Figure 1). One dental school taught implantology in year 1, four of the dental schools taught implantology over 2 years and one dental school taught implantology over 3 years. Five of the schools taught the dental undergraduate programme in consecutive years.

**Figure 2:**
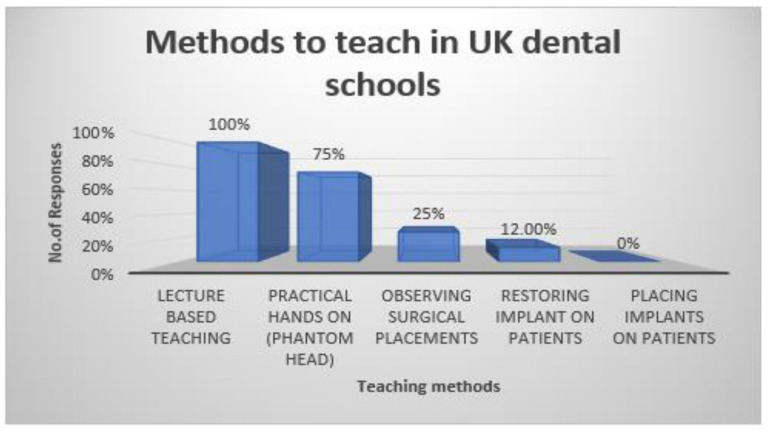
Percentage of teaching methods adopted by the dental schools of the UK.

Of the teaching methods used to deliver the programme, 100% of the respondents had lecture-based teaching (theoretical), 75% engaged students in practical hands-on phantom head work, 25% had observation of surgical placements, and 12% allowed students to restore implants on patients under supervision (Figure 2).

The current teaching aids used to facilitate the teaching of implant dentistry are as follows: 100% of the respondents confirmed that their teaching staff prepared handouts for the students, 62% had a computerised blackboard system in place, 12% of the respondents used DVDs, 25% Implant brochures, 25% Implant manuals, and one dental school stated that their method of delivery was from ‘*learned papers from refereed journals and clinical attachments shadowing in private practice*’. 

Overall, 62% of respondents had a practical element to allow students to engage in hands on placement of dental implants in jaw models/animal cadavers; 12% of responses included an element where students observed implants being restored; 25% allowed students hands-on experience with impression taking for prosthesis on patients; 12% of respondents allowed students to restore implants on patients under supervision; 37% engaged students in restoring implants on models; 12% of respondents allowed students to observe implants being restored on patients; and 12% of the schools allowed students to place implants on patients under supervision. One dental school did not have any practical teaching element in their programme and hence employed an entirely theoretical approach to learning.

Of the 50% of dental schools that participated in the survey, 87% said no implant placement was performed by students. However, one dental school (12%) did allow students to place dental implants under supervision (Table 1). This particular dental school allowed placement of two implants in the anterior mandible which were not connected and were restored with an implant overdenture. 

The topics covered by the lecture programme have changed since the study by Blum (2008). Implant patient education was present in teaching curriculums in 75% of respondents (30%, Blum 2008). Teaching of immediate implant loading was present in 37% of respondents (30%, Blum 2008). The topic of screw-retained vs cement-retained prosthesis moved up from 38% (Blum, 2008) to 75%. Occlusion in dental implants was covered less than previously and dropped from 61% (Blum 2008) to 50% in the current study. Craniofacial application of implants dropped from 53% (Blum 2008) to 37%. Implant post-surgical care dropped from 61% (Blum 2008) to 50%. Implant surgical complications and management rose from 69% (Blum 2008) to 75%. The topics of failing implants, implant prosthetic complications and management moved up from 46% (Blum 2008) to 87%. Topics related to current research and developments in dental implantology changed from 46% (Blum 2008) to 62% in the current study.

### 3.1. Facilitators and Barriers for Dental Implantology Teaching

The implant systems used at the participating universities included Nobel, Straumann and Astra Tech. One dental school reported use of all systems, while another reported that their teaching of implantology was not based on specific implant systems. One participant in the survey said that they did not teach the use of any implant systems. Another school did not accept any help from implant companies due to the NHS not allowing them to do so. Hence, this school must use its own funding for materials, models and teaching aids. The concern raised in this response may be due to the risk of bias developing in teaching programmes, especially if only one implant company was providing support to dental schools.

Support to facilitate the teaching of implantology at UK dental schools varied; however, the universities responded to this question stating a general theme of ‘expertise’ which is afforded by the lecturing staff. One response *was ‘A number of implant-trained teachers, with support from NHS Consultants. A specific patient base. Support from companies delivering content’*. Another dental school replied with ‘*staff expertise and industry*’ suggesting the same. One response stated, ‘*Consultant level staff teach implants in restorative dentistry*’. One faculty stated that they have ‘*Specialist and General Practitioner resources*’ to facilitate implantology teaching at the undergraduate level. Another stated that they ‘*have good staff and space*’. The final stated ‘*Don’t understand the question. I’ve already answered 3 h of seminar with hands-on models*’. Despite universities having staff expertise in the field of implantology, only 25% of the schools that responded to the survey have a structured training programme to prepare lecturing staff for dental implantology teaching.

### 3.2. Training Staff

With respect to qualifications required to teach implantology at their specific dental schools, a wide range of responses were generated, which included the following quotes: ‘*Undertaking or having completed specialist training in fixed and removable pros*’; ‘*Relevant clinical experience or Diploma (Masters level)*’; *All taught by consultants in Restorative Dentistry*’; ‘*Consultant in restorative dentistry actively providing implantology*’; ‘*BDS, PGCert, Specialist Register*’. Three of the eight responding schools required no specific qualification to teach implantology other than the bachelor’s degree (BDS). The GDC has confirmed that, without further training, UK-qualified general dental practitioners would not be able to place implants [15].

With regard to training programmes within dental schools for lecturing staff, two schools had no training at their dental institution for their teaching staff. Other responses included ‘*Training requirement is delivered CPD (in house) and ongoing clinical experience*’; ‘*Mostly done* via *being a StR for the majority. Done over the latter part of the training*’; ‘*Specialist training or on a specialist list*’; ‘*variable*’; ‘*See GDC PfP 2015 LO’s (General Dental Council Preparing for Practice Learning Outcomes 2015) 1.14.12 Recognise and explain to patients the range of implant treatment options, their impact, outcomes, limitations and risks. 1.11.8 Describe the risks related to dental implant therapy and manage the health of peri-implant tissues. 1.14.12 Recognise and explain to patients the range of implant treatment options, their impact, outcomes, limitations and risks*’. Off the respondents, 100% confirmed that patients made no fee contribution to the dental implant treatment provided.

### 3.3. Barriers to Dental Implant Education

Out of all responding dental schools, 75% highlighted cost as a barrier, 37% indicated limited time in the curriculum and 37% selected liability insurance as a problem restricting the implant teaching programme.

Off the respondents, 87% did not receive any financial support from the implant companies, but there was one school that did.

**Figure 3:**
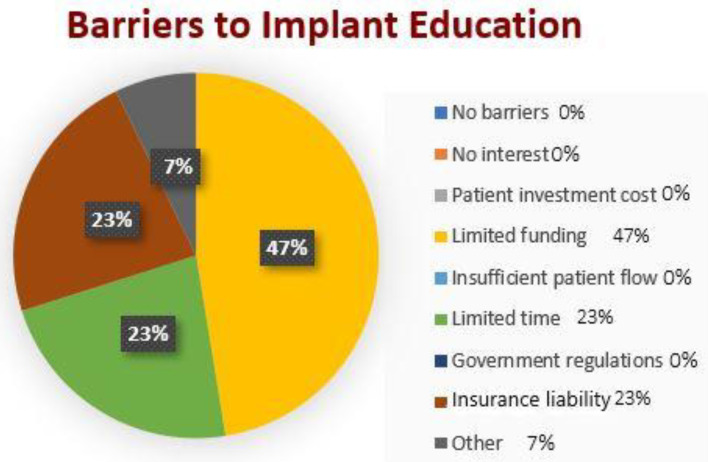
Barriers to undergraduate dental implantology education across the UK.

The study showed that only 25% of the schools that responded to the survey have a structured training programme to prepare lecturing staff for dental implantology teaching, even though there is a norm to which anyone who teaches implantology must adhere (Figure 3).

With regard to any planned changes in the undergraduate teaching programmes of UK dental schools in recent years, one school responded with ‘*Plans for an SSM prior to COVID but put on hold*’. There was no definition of SSM; however, it could refer to ‘soft systems methodology’ which is a tool that helps team-teaching teachers conquer obstacles in collaborative teaching. The implementation of SSM in the team-taught classroom can aid practitioners in achieving Team Learning by inspiring systematic change [15]. Or SSM could be an abbreviation for Students Solutions Manual (Acronym 24). Another school thought of introducing an extracurricular shadowing programme in private dental practices providing implant treatment. This would provide 12 students per year with experience of implantology in general practice. Response 3 explained that their institute is ‘*always keeping up to date with latest peer-reviewed information e.g., 2017 world classification in perio and implants. Clinical teaching staff are perio specialists with strong links to BSP Council and EFP Executive Committee*’. Response 5 was ‘*Practical training has increased. More focus on case selection, managing risk factors and consent*’ Response 6 explained ‘*increased practical component and teaching alongside H&T students to build on teamworking*’. Responses 6, 7 and 8 had no changes and Response 7 went further to explain ‘*GDC PfP was updated in 2015 with no amendments since*’.

GDC regulations and policies were also regarded as a barrier. Direct quotations from the respondents were ‘The NHS does not accept help from implant companies’ and ‘GDC LO’s need to change to help us direct resource to allowing students to treat patients for dental implantology’.

In summary three key findings were identified from the results of the questionnaire. These were developed through coding of the open-ended responses and considering the entire data set. Using Braun and Clarke’s stages of thematic analysis [14,15], the data was coded to identify the key points.

## 4. Discussion

The results of the study confirmed that all dental schools in the UK have teaching of implantology within the dental undergraduate curriculum. The majority of implantology teaching takes place in years 4 and year 5 across the UK dental schools, and 62% of the respondents teach implantology consecutively over 2 years in the undergraduate course. It is essential for the retention of knowledge, as was demonstrated by the i.lect programme from 2008, which concluded that an organised learning programme is very important and aids students in positive learning and information retention, in comparison to an interrupted teaching programme [15]. Off the respondents, 50% taught implantology in the final year. As an advanced subject, it is perhaps more fitting to deliver implantology at this stage of the undergraduate programme, when students have established and formed a basic knowledge of dental science. This will be more relevant as students will have already developed basic practical skills and perhaps will be better equipped to understand and implement this treatment modality.

With respect to retention of knowledge, three out of the eight schools had no form of assessment of the teaching of implantology. The five schools which had assessments varied between informal class tests, summative and formative assessments, or a combination of the three. Assessments were straightforward and helpful in identifying areas of weakness, so increasing information retention [16] and should be part of the implantology programme in the UK in order to solidify knowledge retention. The GDC perhaps should include assessments in the learning outcomes for newly qualified graduates.

Balanced teaching approaches were used to deliver the curriculum in order for students to obtain experience and observation, which strengthens and reinforces what has been learned in the lecture programmes [17].

The results of the current study showed that the hours dedicated to the implant teaching programme varied from 3 h to 25 h, with a mean average of 11 h. Comparing the current study with that of Koole and Bruyn (2014) [7], the current average hours of implant undergraduate teaching in the UK are still less than in mainland Europe, which saw an increase to 74 h in 2014 from 36 h in 2008. 

The results of this study showed that the conservative and prosthetic departments of one university are making changes to their curriculum to include an implant dentistry subject. This indicated that the dental school in question was planning to expand its implantology curriculum. An evaluation of the curriculum and learning outcomes suggested that implantology is becoming more relevant in undergraduate education. Some schools also planned to add clinical attachment courses and shadowing in private practice to increase teaching and student experience in implantology. This suggests that dental schools are seeking to improve learning methodologies. Private practice shadowing also implies that they value implantology as a discipline. The results also showed that the emphasis of practical education was on model placements rather than restoring implants. This may be due to the high cost of implant prosthesis and possibly the dental schools’ lack of qualified technicians to manufacture implant prosthesis. This practical element was reported by Chin et al., (2018) [8], where 88% of the respondents confirmed that this was the most taught format used in the undergraduate teaching programme. This differed from the current study, where 100% of respondents reported lecture-based teaching being the most prevalent method. There was a difference between schools permitting impression taking for prosthesis (25%) and schools allowing implant placement (12%), possibly because it is a more controlled operation with less risk to the patient. This is in line with the study by Chin (2018) and demonstrates that no real change has taken place since that time with respect to surgical placements [8].

In this study, all participants agreed that teaching dental implantology is important because it is adequately covered in their undergraduate curriculum. One dental school admitted that their curriculum did not fully address dental implantology, but that it was currently under review. With respect to the study by Blum in 2008, there has been a marked increase in certain subjects taught at the undergraduate level. According to the study’s findings, undergraduate instruction is relatively broad and comprehensive, with modest decreases in some topics, maybe due to low participation. The following subjects saw the most changes: ‘Implant patient education’; a rise of 20–30% in growth in 2015 may be related to implantology becoming a more mainstream technique to replace teeth [18]. ‘Screw retained vs cement retained restorations’; this may be owing to the evolution of implantology and the necessity for retrievability in restorative repair and maintenance [19]. ‘Failing implants, complications, and management’; as the number of placements has increased, so has the number of difficulties, making situation management and preventing complications critical. Thus, subject content appears to be more uniform and balanced throughout UK dentistry schools.

The general impression is that the faculties are training their students to be safe beginners who understand the concepts of implantology and possess specific implant knowledge as described by the GDC. Additionally, dental implantology is considered to be an elective, advanced discipline; the aim of the undergraduate programme is to make sure that newly qualified dentists know all the options so that they can make the appropriate referral. The limitations to the dental curriculum in implantology appear to be driven by achieving the basic requirement as described by the GDC [20].

In the closed questions, 75% of responders (six of eight schools) indicated ‘Cost to Dental Hospital (limited funding)’ as a barrier. In the 2018 study by Chin, Lynch et al. [8], 12 out of 16 institutions (75%) selected finance as the greatest obstacle to providing implant training at the undergraduate level, and 9 out of 16 respondents (56%) identified time within the curriculum as a major factor restricting the provision of teaching. In the current study, 37% of respondents said this was a difficulty. 37% of the respondents said ‘liability insurance’ was a burden to implant teaching. Chin et al.’s investigation did not identify this as a barrier (2018).

One dental school reported that they received funding from dental implant companies. Thus, implant companies are willing to provide financial aid. Dental schools should explore this avenue, as finances and funding seem to be the most prominent barrier to the progression of the undergraduate implant programme. If implant companies and dental schools form partnerships, there is a risk of bias and commercialisation arising in dental educational programmes. So, there must be a balance. If implant companies are willing to help, their lecture content must be carefully reviewed. Forming partnerships with multiple companies may help decrease bias in teaching. According to one dental school, implant companies already supply learning aids such as models and educational materials. The limited time within the curriculum is still an issue, though some dental schools are reviewing their curricula. Some schools will just deliver the GDC’s minimum requirements. However, most schools (62%) had recently changed their curriculum to include more practical experience and shadowing opportunities for students. This shows there is curriculum review taking place amongst dental schools even without changes in the GDC guidelines. Patient pool and case selection is important and was not identified as an issue/barrier to providing dental implant treatment. McAndrew and Ellis (2010) [21] suggested that advanced clinical training should be limited to a select group of students who are highly motivated.

To conduct an effective teaching programme, instructors must be properly trained. This would imply the curriculum considers the necessity for adequately trained staff to deliver the programme. The current survey found that only 25% of respondents have established programmes to train teaching personnel in dental implantology. This is in accordance with the findings of Chin et al., (2018), who identified teacher shortages as the third most common issue.

There appears to be a consensus that more training beyond a bachelor’s degree is required to teach implantology to students. This view is reinforced by the phrases “specialist”, “expertise” and “consultant”. For teaching implantology, three dental schools reported that a bachelor’s degree in dental surgery was sufficient. Hendricson et al. (2007) [13] showed that a professional development programme based on a train-the-trainer methodology was shown to strengthen the capability of lecturing staff to provide implant education to dentistry students [20]. This would resolve the issue of there being limited trained staff in UK dental schools to provide implant teaching and ensure that faculties are not so reliant on recruiting new staff, as they will be developing expertise in-house.

This finding may hinder progression and development of implantology undergraduate teaching, but may improve if the ‘*GDC learning outcomes change and help direct resource to allow students to treat patients for implantology*’ as stated by one dental school in response to the questions of barriers to dental implant treatment at their institution.

The GDC has confirmed that general dentists in the UK cannot place implants without additional training [13]. The teaching staff of these schools may have received some training, but not a postgraduate qualification. Due to their lack of knowledge, students may be unable to place or observe dental implants. Chin (2018) found that 4 of 16 respondents identified ‘limited numbers of appropriately educated professionals’ as a barrier to undergraduate teaching [8]. Therefore, the practical teaching programme may be limited due to a lack of trained staff and funding.

Institutional training programmes to prepare staff for the teaching of implantology appeared haphazard. The GDC may be dictating the training programme’s limit, as some organisations will only do what is required of them. One respondent thought the question was vague, but claimed that the teaching is performed by appropriately qualified personnel, without specifying how this was done. Curriculum congestion was identified as a barrier by McAndrew, Ellis et al. in 2010 [21]. This issue appears to be improving due to dental schools reviewing their current curricula and trying to increase modular content by introducing shadowing programmes.

Curriculum limitation is dictated by the GDC: this has been emphasised by respondents to the question regarding barriers which exist to the progression of dental undergraduate implantology. The emphasis from the GDC is to ensure the newly qualified graduate understands the concepts of implantology, but is by no means able to place dental implants upon graduation. If the GDC were to review the learning outcomes in their ‘Preparing For Practice’ document, then UK dental schools would have to modify their curricula in line with the changes.

In summary, the curriculum content of the undergraduate programme is aligned with the GDC recommendations [22], but some schools are going beyond the basic requirement of providing comprehensive training for beginner level, to understanding of concepts. Practical and theoretical teachings are present, but they appear to be more focused on lecture programmes and phantom head work. Funding, restricted time and in-house staff training programmes are barriers to the development of the implantology teaching programme.

An adequate response rate is required to generate a significant research conclusion. Bigger response rates yield more accurate results. This study’s sample size is 16. Because this is the total number of dental schools in the UK, it may be difficult to draw meaningful conclusions from the data. Statistical studies usually necessitate a greater sample size to ensure that the sample is representative of the population and that the statistical finding can be applied to a larger group [23].

The response rate may also be influenced by the pandemic, which has left universities understaffed due to the furlough programme and subsequent redundancies. As a result, faculty priorities may have shifted, affecting research projects or questionnaire replies. [24]. A follow-up phone call to the School Office may have drawn their focus to the letter of invitation, encouraging them to assign it greater priority. This may have increased the response rate [25]. Some of the responses stated that the questions were unclear, thus future work may benefit by piloting with a number greater than three, as in the current study, with grouping of questions of comparable topics together under subheadings.

The findings of the study showed that there has been an improvement and change in undergraduate teaching of dental implantology in the UK. All respondents confirmed that there is teaching of dental implantology taking place at the undergraduate level. There is more uniformity in the subject matter taught in the lecture programmes. However, the hours dedicated to teaching the programme are still less than the time allocated across the rest of Europe. The universities appear satisfied with their curriculum content, but this is somewhat limited by the GDC, as some schools do not go beyond the GDC’s recommended guidelines. Funding is the most prevalent barrier to progression in dental implant teaching. Curriculum review was a finding which suggests there may be changes ahead with regard to increasing the time allocation dedicated to implant dentistry, but the limiting factor here may be suitably trained staff to deliver the programme. In-house structured learning programmes are limited to a few schools. This could lead to a bottle-neck situation and dental schools should look at ways to train staff in-house, to ensure there is never a skills shortage when staff leave. Forming partnerships between implant companies and dental schools may help, as funding may be provided, as identified by one dental school who responded to the survey. However, the schools must be careful as the teaching programmes may end up biased towards particular companies, hence partnerships with multiple implant providers should be pursued. How teaching is conducted should be investigated, with a specific focus on how time is divided between practical and theoretical learning. A further investigation is required to see if any informal teaching takes place. An improvement and change in undergraduate teaching of dental implantology has been observed in the UK, with greater uniformity seen in the subject matter taught in the lecture programmes.

The hours dedicated to teaching the programme varied from 3 h to 25 h, with a mean average of 11 h. This is still behind the time allocated for the teaching of dental implantology across mainland Europe. Additionally, dental implantology is an elective, advanced discipline; the aim of the undergraduate programme is to make sure that newly qualified dentists know all the options so that they can make the appropriate referral. The limitations to the dental curriculum in implantology appear to be driven by achieving basic requirements as described by the GDC.

## 5. Conclusions

Universities should report on their implant programmes in detail so that they may be compared and replicated in other settings, allowing for further progress. Perhaps a forum should exist uniting the dental schools of the UK to help facilitate such progression. Ultimately the GDC is dictating the dental schools’ curricula and there have been no real changes in their learning outcomes since 2002. Hence, if implantology is to progress at the undergraduate level, there must be changes to the learning outcomes dictated by the GDC to prepare General Dental Surgeons for practice. Gaining students’ perceptions is important, to see how they feel about their implantology teaching programme and if they would like to see any changes. It would also be interesting to approach implant companies and ask their views on partnerships between themselves and dental schools in the UK. Finally, perhaps dental schools should be asked what they feel should or could be done in their institutions to remove/reduce the barriers to progression of the implantology teaching programme. 

## Figures and Tables

**Table 1:** Practical teaching of implant dentistry amongst undergraduate dental students.

Format of Practical Teaching in Implant Dentistry	Responses	
	Number of Responses	Percentage%
Students observing implant placement	1	12
Students observing implants being restored	1	12
Hands on placement in jaw models/animal cadavers	5	62
Hands on experience with placing implants under supervision	1	12
Hands on experience with impression taking for prosthesis on patients	2	25
Hands on experience with restoring implants on patients	1	12
Hands on experience with impression taking for prosthesis on models	3	37
Hands on experience with restoring implants on models	3	37
No practical teaching	1	12
Other	0	0

## Data Availability

Not applicable.
